# A deep learning approach to estimating initial conditions of Brain Network Models in reference to measured fMRI data

**DOI:** 10.3389/fnins.2023.1159914

**Published:** 2023-07-17

**Authors:** Amrit Kashyap, Sergey Plis, Petra Ritter, Shella Keilholz

**Affiliations:** ^1^Berlin Institute of Health at Charité – Universitätsmedizin Berlin, Berlin, Germany; ^2^Department of Neurology with Experimental Neurology, Brain Simulation Section, Charité – Universitätsmedizin Berlin, corporate member of Freie Universitat Berlin and Humboldt-Universitat zu Berlin, Berlin, Germany; ^3^Department of Computer Science, Georgia State University, Atlanta, GA, United States; ^4^Wallace H. Coulter Department of Biomedical Engineering, Georgia University of Technology and Emory University, Atlanta, GA, United States

**Keywords:** Brain Network Model, fMRI, deep learning, Neural ODEs, initial condition

## Abstract

**Introduction:**

Brain Network Models (BNMs) are mathematical models that simulate the activity of the entire brain. These models use neural mass models to represent local activity in different brain regions that interact with each other via a global structural network. Researchers have been interested in using these models to explain measured brain activity, particularly resting state functional magnetic resonance imaging (rs-fMRI). BNMs have shown to produce similar properties as measured data computed over longer periods of time such as average functional connectivity (FC), but it is unclear how well simulated trajectories compare to empirical trajectories on a timepoint-by-timepoint basis. During task fMRI, the relevant processes pertaining to task occur over the time frame of the hemodynamic response function, and thus it is important to understand how BNMs capture these dynamics over these short periods.

**Methods:**

To test the nature of BNMs’ short-term trajectories, we used a deep learning technique called Neural ODE to simulate short trajectories from estimated initial conditions based on observed fMRI measurements. To compare to previous methods, we solved for the parameterization of a specific BNM, the Firing Rate Model, using these short-term trajectories as a metric.

**Results:**

Our results show an agreement between parameterization of using previous long-term metrics with the novel short term metrics exists if also considering other factors such as the sensitivity in accuracy with relative to changes in structural connectivity, and the presence of noise.

**Discussion:**

Therefore, we conclude that there is evidence that by using Neural ODE, BNMs can be simulated in a meaningful way when comparing against measured data trajectories, although future studies are necessary to establish how BNM activity relate to behavioral variables or to faster neural processes during this time period.

## 1. Introduction

Brain Network Models (BNMs) represent whole brain activity as the coordination of many distinct neural populations that are connected via a structural network consisting of long-distance white matter tracts (Sanz-Leon et al., 2015; Breakspear, 2017). Simulations of these network models are being compared to experimental measurements such as functional magnetic resonance imaging (fMRI). At this spatiotemporal scale in fMRI, the measured activity is thought to be an averaged property of the neural populations and occurs relatively slowly (<1 Hz) compared to the faster neural information processes and the measured fMRI signal is thought to represent the coordination between brain regions over the structural network (Deco et al., 2009). BNMs have been able to reproduce properties observed in fMRI, especially during rest where the brain is not exposed to a structured experimental task or stimulus and the whole brain activity is thought to mostly arise from intrinsic network loops between cortical regions (Honey et al., 2007; Cabral et al., 2011; Sanz-Leon et al., 2015). Thus, BNMs have been used as a generative framework in order to analyze how local neural activity could translate to global coordination and how changes due to neural pathologies translate to observed aberrant dynamics (Ritter et al., 2013; Sanz-Leon et al., 2015; Saenger et al., 2017; Schirner et al., 2018).

Current research does not utilize a single type of population model to construct BNMs. Instead, depending on the application and the underlying assumptions, different neural mass models are selected to represent whole brain activity (Sanz-Leon et al., 2015). For replicating rs-fMRI, several models have been shown to reproduce time-averaged properties such as functional connectivity (FC), computed via cross correlation of long time-courses of pairs of brain regions (Cabral et al., 2017). Due to the lack of a stimulus onset used as a reference in rs-fMRI, comparisons between simulations and measured data are made over a long time window and use time averaged metrics rather than direct comparisons of the predicted trajectories with the measured timeseires (Cabral et al., 2011, 2017; Kashyap and Keilholz, 2019). Researchers are interested in BNM predictions on a time-point basis because many neural processes observed in fMRI occur over these timescales, such as responses to task stimuli or aberrant responses due to neural pathologies. While previous studies have examined faster processes in BNM’s, such as comparing them with multimodal recordings such as EEG data (Schirner et al., 2018), no prior investigations have examined how well short-term trajectories, defined by a series of consecutive fMRI measurements, are being reproduced by current BNMs. To address this gap, we solve for initial conditions relative to an observed trajectory for a given BNM and then compare the synchronized predictions of the simulation with the observed timeseries.

We hypothesize that the BNMs that are better approximation of the underlying dynamical system in whole brain dynamics determined using traditional long-term measures, will evolve more closely to the measured rs-fMRI trajectories. An agreement of parameterization between the long-term metric and the investigated short term metrics would support the evidence that BNM’s are simulating meaningful trajectories. Moreover with these initial conditions, BNMs can be later used to simulate and investigate neural processes during these short timeframes such as relation to task fMRI behavioral variables.

The initial conditions are estimated, by utilizing a novel method developed in the Machine Learning community that utilizes a sequence of observations and a given dynamical system to output the initial conditions of the dynamical system that would be the closest fit to the current observed data trajectory. The technique, known as Neural Ordinary Differential Equations (ODE), uses a recurrent neural network (RNN) that keeps track of information from previous timepoints, in order to predict the initial conditions of a given dynamical system based on previous observations (Chen et al., 2019). The neural network model is trained via one step prediction, namely from the estimated initial conditions we integrate the known dynamical system to predict the next timestep and compare it with the true next step. The algorithm, therefore, regardless of the dynamical system, gives similar predictions over the first-time interval but the trajectories diverge over longer periods of integration due to differences in the dynamical system and become less dependent on initial RNN predictions.

A potential issue to this approach is that both the signal simulated as well as the measured rs-fMRI signal are thought to be produced by stochastic processes. For simulations this is achieved by adding noise to the models differential equations. This noise might affect the approaches’ ability to discriminate correctly between different BNMs on their ability to simulate rs-fMRI, as it adds variance to the data. However, previous studies have shown that despite their variability dynamic metrics are better than metrics computed over a long period of time to parameterize BNM, thus suggesting that even in shorter windows allows for discrimination between models (Kashyap and Keilholz, 2019).

To test whether this approach can correctly identify components of a known BNM, we used the Firing Rate Model (FRM) from Cabral et al. (2012), as a candidate dynamical system to fit to the rs-fMRI data. The FRM is a linear model that defines the change in dynamics in a single neural population as a weighted sum of its network neighbors and applies an exponential decay term to prevent runaway excitation (Cabral et al., 2012). The model contains three components (global coupling, noise amplitude, structural matrix), which are varied independently, and a specific Neural ODE is trained for each variation to solve for the initial conditions. The results show that without noise, maximizing accuracy over the short time window yields trivial BNMs that do not depend on the structural connectivity. However, in the presence of noise the trend reverses and models with strong structural connectivity perform better than the models with weak or no network influence. Since the value of noise is unknown, an additional parameter, namely the structural connectivity was varied by slowly adding noise to the original connectivity, and the sensitivity due to this change in network was measured. The FRM that exhibited the greatest changes in accuracy due to perturbations of the structural connectivity had a parameterization that was in agreement to the one established using long term metrics.

In short, the manuscript demonstrates that Neural ODE approach can simulate FRM trajectories that can be meaningfully compared with measured rs-fMRI data. The differences in parameterizations of the model with respect to rs-fMRI data observed during this timeframe are similar to those observed over longer simulations. Therefore, this tool can be used in the future to analyze BNM on shorter timescales with respect to measured data such as for task fMRI. Furthermore, it can serve as an unbiased metric to directly compare the signal with the models and aid in the development of discovering more powerful models that recapitulate whole brain activity.

## 2. Methods

### 2.1. Overview

This section is organized by first describing functions that are used to fit to the rs-fMRI data, mainly BNMs but also certain null models that are used for comparison. The subsequent sections then describe how the Neural ODE algorithm is used to infer the initial conditions of a given dynamical system based on previous measurements. We describe our own implementation of the Neural ODE algorithm that was specifically designed to train on large amounts of imaging data (Chen et al., 2019). The algorithm was validated using synthetic data from a simple spiral dynamical system described in detail in the Supplementary sections. The subsequent sections after describing the algorithm, deal with the processing of experimental fMRI and DTI data used to construct the models. The final section outlines how the simulated trajectories are compared with empirical trajectories.

### 2.2. Brain Network Models

Brain Network Models are used as models for whole brain network activity. BNMs combine a mathematical description of the intrinsic activity of a neural population with the global brain structure that coordinates the activity between populations. To construct a BNM, researchers first define a structural network, based on a parcellation scheme that outlines which cortical areas work cohesively as a neural population. In this manuscript, the Desikan Killiany atlas is used as a parcellation scheme as it has been used successfully before for whole brain simulations (Cabral et al., 2011, 2012). Only the cortical areas without the insula are represented in the model constituting a total of 33 regions for each hemisphere for a total of 66 brain regions. These regions serve as the nodes in the network model, while network neighbors are defined using tractography to map out fibers that connect two regions of interest. The change of the activity in the ith brain region is defined as follows:


(1) $$\begin{array}{l} \dot{x_{I}}=\sum_{J\in \mathrm{Neighbors}\;\mathrm{of}\;I}F\left(x_{I},x_{J},\rho_{IJ}\right)\\ \;+\sum_{K\in \text{Task inputs}}G\left(u_{K},{À}_{KI}\right)+N\left(0,Ã\right) \end{array}$$

The first term represents the network component which is described by a function F that depends on its own activity *x_i_*, activity in its neighbor *x_j_*, and the physical properties of the fiber represented by the vector ρ (i.e., the number of fibers between regions, the delay in propagation). The second term consists of a function G that represents external input, whose activity is represented by a k-dimensional vector *u* representing all sub-cortical and sensory inputs, and the vector π representing again the physical properties that project these inputs into the cortical model (i.e., thalamic tracts into cortex). The last term represents a zero-mean Gaussian noise from the neuronal populations or from omitted higher order terms from the network equations. For resting state activity, the assumption is that $u_{k}$ *(t) = 0 ∀ t* and the first term dominates the change in activity. This still leaves a large family of functions that are used to approximate *F*, with many parameters that can widely change the dynamics of the system. In theory, all of these functions can be used to fit the fMRI data with the Neural ODE algorithm, and for each of them initial conditions can be estimated from empirical data. In this manuscript, we focus on the Firing Rate Model, the simplest model that can recapitulate whole brain activity, and use it to solve for initial conditions in the Neural ODE.

#### 2.2.1. Firing Rate Model

The FRM represents the activity of a brain region as the mean firing rate. The change in firing rate of a region depends on a weighted sum of all its neighbors’ activity (Eq. 1). The FRM has two parameters: the global coupling parameter *k*, which controls the strength of network input, and the level of noise amplitude σ, which simulates random activations of brain regions due to unknown neuronal activity (Cabral et al., 2012). At values of *k* < 1/(max eigenvalue of *W*), the system is stable and the system decays to the origin without extraneous noise input. Typical values are *k* = 0.9/(max eigenvalue of *W*) and σ = 0.3 (based on the Desikan Killiany atlas) where there is a trade-off injecting noise in order to perturb the dynamics and the relative strength of the network to keep the neural areas functionally linked over time (Cabral et al., 2012).


(2) $$\dot{x_{I}}=-x_{I}+k\sum_{J\in \mathrm{Neighbors}\;\mathrm{of}\;I}W_{IJ}*x_{J}+N\left(0,\sigma \right)$$

#### 2.2.2. Parameter and structural perturbations

Each of the components of the FRM are varied. These components include the global coupling parameter *k*, the amplitude of the noise level σ, and the structural weight matrix *W*. The global coupling parameters and the structural matrix are fixed before using the Neural ODE algorithm, such that for each specific set of values for *k* and *W* a separate LSTM network is trained to generate initial conditions. To vary the global coupling parameter, the parameter *k* in Eq. 1, is adjusted from 0.9 to 0 in 0.15 intervals. To generate structural perturbations, a random percentage of the original edges are swapped to connect two different nodes while keeping the graph symmetric. This creates random perturbations from the original structural matrix while maintaining the number of edges. Each of these graphs would result in different dynamics, but the trajectories from the model containing the original structural connectivity should be the closest to the measured rs-fMRI data. The noise parameter is not used in training the LSTM Neural ODE model. However, during testing after the initial conditions are estimated the noise is introduced by testing σ using values [0.0001, 0.15, 0.3, 0.45].

#### 2.2.3. Null models

To compare the effects of fitting to the Neural ODE with other functions, the rs-fMRI data is fitted with null models that do not simulating network activity. The simplest of these models sets the Neural ODE equations to 0, such that the prediction of the LSTM is the output of the model. This quantifies how well the LSTM network’s initial condition prediction matches the next predicted output without any of the BNM functions. For future timesteps, it acts as a simplified autoregressive model by holding the current input as the output, i.e., *x(n + 1) = x(n)*. This is implemented by setting the connectivity matrix in Eq. 2 to an identity matrix which cancels out with the first term and sets the equation to 0, and is referend to as the Autoregressive (AR) model. The second null model is obtained by setting the global coupling parameter to zero in the FRM and the differential equations reduce to an exponential decay. This model should perform worse than the BNM equations but test the limits of the global coupling values. Finally, we compare it to a pure Machine Learning Inference model as published in Kashyap and Keilholz (2020), where at each timestep, the output of the LSTM is fed in as the next input. This model is non-deterministic as the output of the LSTM is sampled from a distribution. The function implemented by the LSTM in this case is completely unknown, as even the noise level changes as a function of the input. This model, however, gives a good estimate of an upper-bound on predictability on a short time window and does better than using traditional BNMs and can replicate complex resting state processes as Quasi Periodic Patterns and dynamic functional connectivity analysis performed using K-means (Kashyap and Keilholz, 2020).

### 2.3. Neural ordinary differential equations

**Figure 1:**
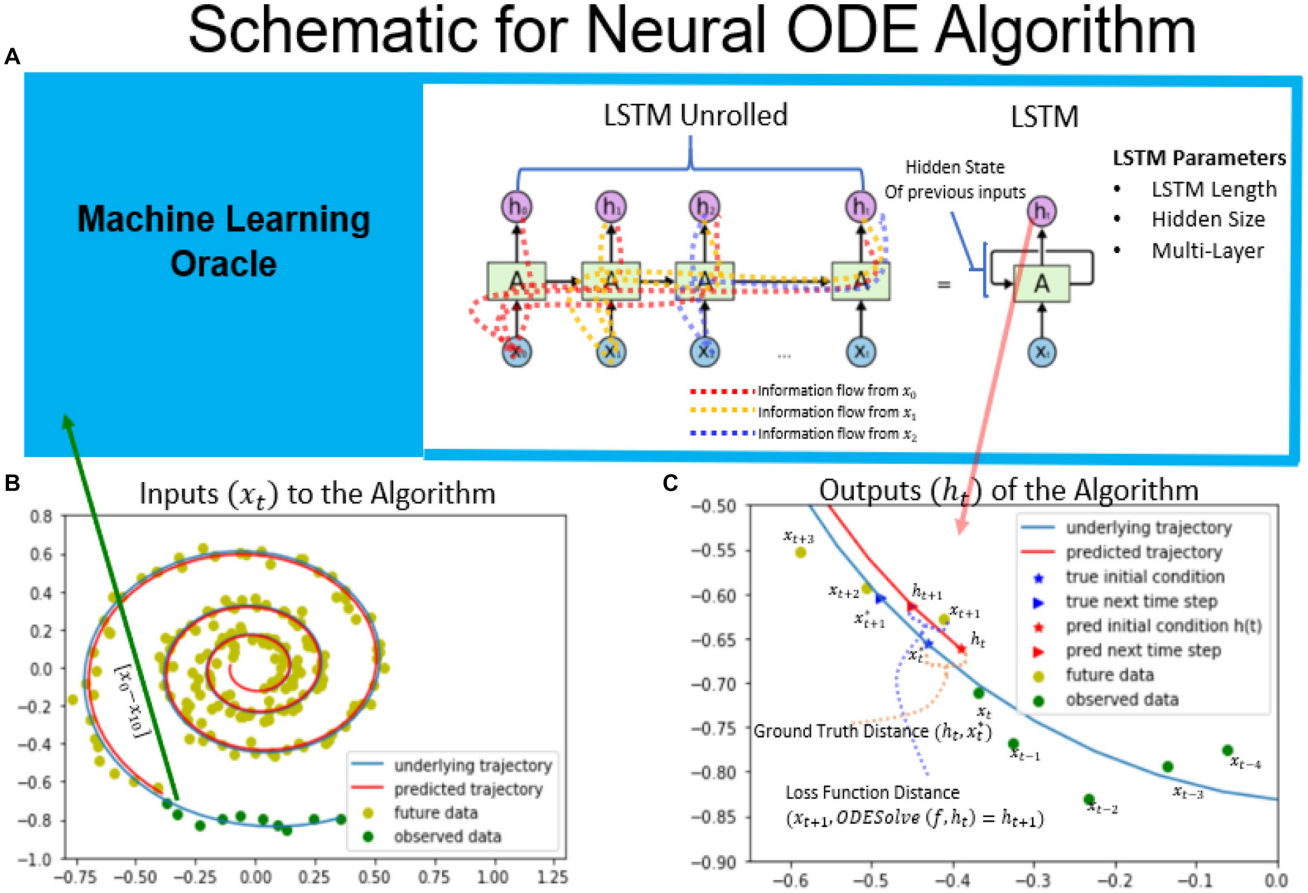
Schematic of the neural ODE algorithm. Schematic of the neural ODE algorithm. An example spiral trajectory is shown in panel **B** with green points representing the data sequences. The RNN takes in one data point at a time and updates its hidden state as well as outputs its prediction for the initial condition. The hidden state keeps track of information from previous observations and is carried forward to future time steps, as illustrated in the RNN unrolled diagram. The output of the RNN represents the initial condition of the dynamical system at that timestep. For the spiral dataset, the true ground truth initial condition $x_{t}^{*}$ is known and is illustrated in bottom right. The ground truth distance is used to evaluate the trained RNN network’s ability to predict the initial conditions in the constructed dataset. The next timepoint is predicted by integrating the ODE system based on the given dynamical system. The loss function is defined as the difference between the next predicted timepoint and the next observed time point, $x_{t+1}$. Minimizing the loss function distance minimizes the distance to the ground truth initial conditions. (Mars, 2020).

The Neural ODE algorithm is designed to estimate the initial conditions of a given dynamical system based on past observations. Figure 1 provides an overview of the algorithm, which involves fitting to a spiral dynamical system based on noisy observations using Neural ODE. The task of the recurrent neural network is to predict the true initial conditions of the spiral dataset (shown as blue underlying trajectory in Figure 1) based on the sequence of observed measurements shown in green. A RNN implementation known as Long Short Term Memory (LSTM) was used to perform this task, as it keeps the information of past data observations [*x_0_* to *x_t-1_*] in its hidden state p_t-1_ (Graves and Schmidhuber, 2008). Thus, when the timeseries is fed into the RNN one timepoint at a time, the current information is incorporated into the hidden state and is passed forward as shown in the LSTM unrolled version, in order to aid in the prediction of future observations. The LSTM’s predictions become more accurate as it observes more data, up to a certain limit beyond which newer data does not add any new information to the hidden state (Graves and Schmidhuber, 2008). For this particular task, the LSTM’s output is defined as the initial conditions of a given dynamical system. Since the initial conditions are not known and thus an effective gradient cannot be computed based on initial conditions alone. Therefore, the algorithm assumes that the next observation is the integral of the predicted initial conditions and the given dynamical system with some noise added to it (Chen et al., 2019). The loss function is calculated based on the measurement at the next timestep in ensure the output of the LSTM to converges to the correct initial conditions for the given timepoint. A schematic of the algorithm as well as the Tensorflow implementation are shown in Supplementary sections 7.1, 7.2.

### 2.4. Experimental data

#### 2.4.1. Structural network for Brain Network Model

To estimate the structural network, tractography was run on 5 HCP Diffusion Weighted Images using the freely available software Mrtrix (Van Essen et al., 2013; Kashyap and Keilholz, 2019). The fiber orientations in the DWI images were first estimated using constrained spherical deconvolution. Next, using a probabilistic streamline algorithm, 100 million fibers set at a maximum length of 250 mm were computed for each individual and then filtered to 10 million fibers. To construct the structural network, we determined the number of fibers that intersected two ROIs in the Desikan-Killiany atlas and normalized the power by dividing by the surface area of the receiving region (Desikan et al., 2006; Hagmann et al., 2008; Cabral et al., 2011). Finally, the matrix is normalized by dividing by the largest eigenvalue such that the graph Laplacian (k*SN-I) has only negative eigenvalues (Cabral et al., 2012). This normalization ensures that the feedback decays and prevents an exponential increase in the signal over time.

#### 2.4.2. fMRI data

The fMRI data used to test and train the models were obtained from the Human Connectome Project 447 Young Adult subjects release. The scans were pre-registered to Montreal Neurological Institute (MNI) space in surface format (MSMAII) and denoised using 300 Independent Component Analysis, following the recommended steps by Salimi-Khorshidi et al. (2014). The surface-vertex or grayordinates time series were transformed to the ROI time series by averaging all vertices based on the Desikan-Killiany atlas parcellation. This was done on an individual level since the surface parcellations are provided to by HCP and Freesurfer for each individual subject (aparc and aprac2009 files). The signal was then bandpass filtered from 0.0008 to 0.125 Hz and then the global signal was regressed using a general linear model using the mean timeseries of all cortical parcels as the global signal. Finally, the signal is subsequently z-scored as described in Kashyap and Keilholz (2019). For the task data, each dataset (language, working memory, motor, social, emotional, gambling, relational) was processed separately and then concatenated together. Each task dataset was truncated to the closest multiple of 50 timepoints and the autoencoder was fed alternating segments of task and the rest data for training. The algorithm was trained using both task data as well as rest data, because the algorithm performed better on most metrics with more varied data. Furthermore, it is believed that during task activity, resting state networks dominate most of the cortical activity and task networks often look indistinguishable from rs-fMRI networks (Smith et al., 2009). However, during evaluation, only the results on predicting future rs-fMRI were presented, while task will be addressed in future work.

### 2.5. Metrics and evaluation between simulated and empirical trajectories

The dynamical models are evaluated on how well they fit with the empirical observations from the estimated initial conditions. For the spiral dataset, the true initial conditions were known, allowing for direct calculation of results using a Euclidean distance between the estimated and true initial conditions. For the fMRI data, the r-squared and the mean squared error at each timepoint between the predicted and observed data vectors representing the activity of 66 brain regions were calculated. Since the loss function of the Neural ODE algorithm, converges to zero during training across most models, this metric tends to be most similar when comparing across models (see Supplementary section 7.7). Therefore, in order to differentiate between the models, the error was calculated for subsequent timepoints to gauge how well the trajectory follows the timeseries over a longer period of time. The results were calculated across a set aside test dataset using a batch of subjects (*N* = 80). The Supplementary section 7.5 discusses the differences in variances between group and individual models, and the effect of testing at every timepoint versus a few timepoints. While there is no pronounced difference in testing a few timepoints and evaluating the system at every timepoint, there is a large difference in variance between the averaging the error out in a batch of subjects (*N* = 80) vs. in the individual models. The group metric was selected for its smaller variance and greater robustness, given the purpose was fit a group model rather than an individual model for the HCP resting state dataset using the Neural ODE algorithm.

## 3. Results

The objective of this study was to assess the feasibility of using Neural ODEs to solve for the initial conditions of BNMs, and subsequently differentiate between different models based on how well they follow the resulting rs-fMRI measurements. To validate this approach, the methodology was applied on a constructed spiral dataset where the true underlying dynamical system was known and the correct coefficients are shown to be determined using this approach. Subsequently, the technique is applied to fit with the rs-fMRI dataset using a FRM, and the model parameters and coefficients are estimated and shown to be similar to previous literature.

### 3.1. Differentiating between dynamical systems on spiral data

The process of generating the spiral dataset was explained in detail in Supplementary sections 7.3–7.7, but, in essence, it involved a two variable linear dynamical system and that was often used as an example in machine learning literature to show the feasibility of solving for initial conditions. The Supplementary sections began by showcasing the results from the previous paper Chen et al. (2019), where the Neural ODE algorithm’s predictions converged to the right initial conditions after observing a sufficient number of previous timesteps to allow the LSTM to make valid predictions. The subsequent sections detailed the methodology used to determine the network hyperparameters such as the hidden size and number of layers. Larger networks were found to be more sample-efficient as they could predict the initial conditions based on fewer timepoints. At a certain size, the accuracy did not improve much with alterations to the network and was used as the model to perform the following experiment on system identification.

**Figure 2:**
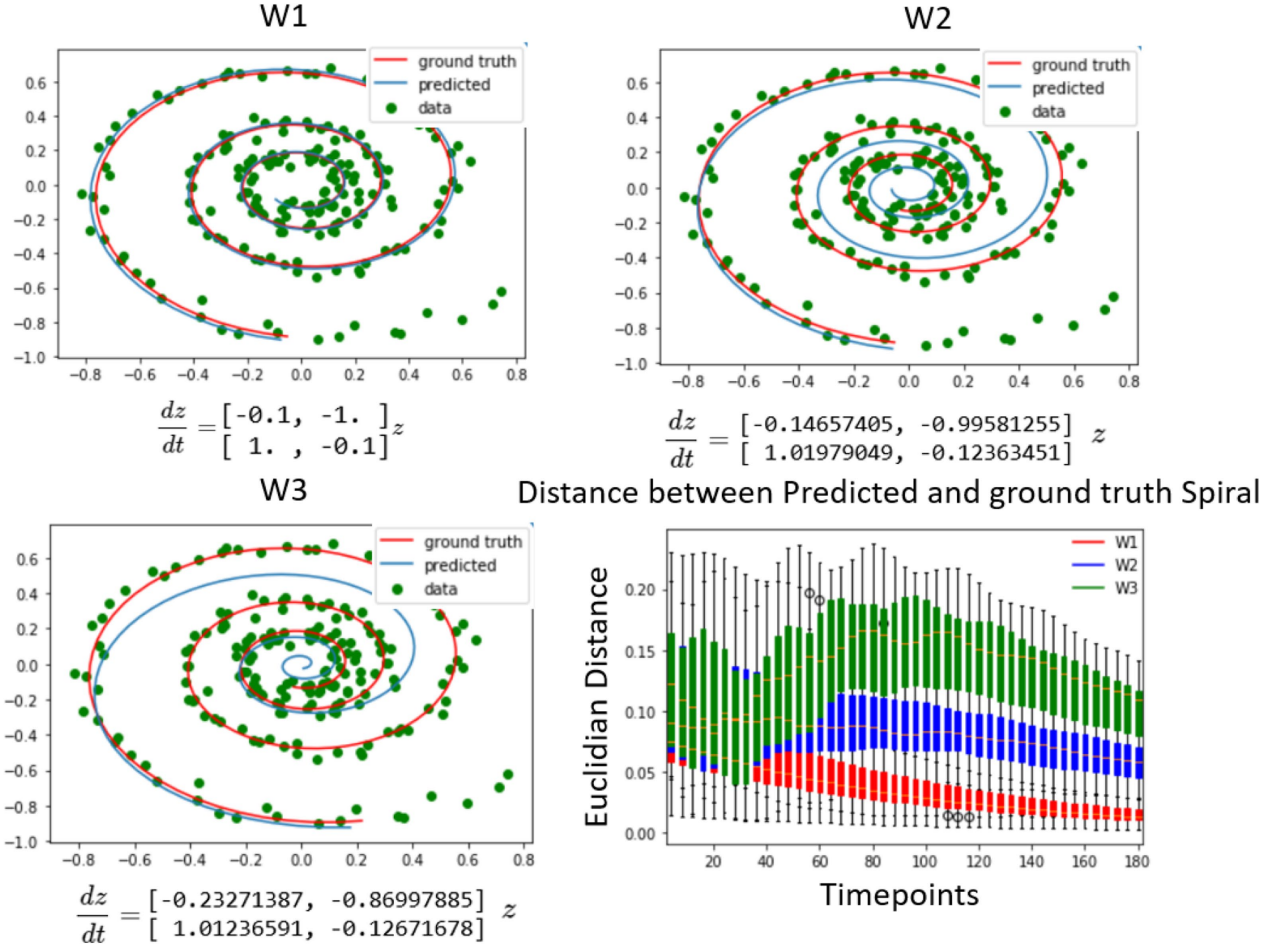
Differentiating between dynamical systems on spiral data. The study utilized a number of candidate spirals, which were a perturbed version of the ground truth spiral, to fit using the neural ODE algorithm to the spiral data. The top left shows a spiral that was well aligned with the data, as it was used to generate the data. The top right and bottom left figures showed spirals that were increasingly further away from the ground truth. The neural ODE algorithm was able to fit any of the spirals to the data for a given set of observations, but over time, the candidate spirals that were further away from the ground truth diverged much faster from the future data points. This divergence was quantified by the plot on the bottom right, where the distribution of the distance between the three different spirals and the observed data points was plotted from the predicted initial conditions. Initially, the distance between the spirals of different weights and the observed data points was close, but it diverged further away when compared to future timepoints. It is noteworthy that due to all trajectories going toward the origin, the distance at very large timescales converged to zero, but this is not expected in the brain data, where the signal does not approach a single attractor. (Mars, 2020).

The spiral data was generated using a known system of differential equations as presented in Figure 2 (top right). The objective of the Neural ODE algorithm was to solve for the initial conditions for each of the candidate dynamical systems with respect to the observed data. Each of the systems contained distinct coefficients in their respective weight matrices, where W1 represented the original dynamical system and W2, and W3 contained the original structural matrix perturbed with increasing noise. The findings illustrated in Figure 2 depicted three instances of fitting these spirals to generated spiral data for different weight matrices. The results demonstrated that, in short time periods after the initial conditions, the other candidate systems fit the data as well as the original system for the first few points after the initial conditions. Nonetheless, in the long run, the spiral matrix W1 was found to be the closest to the data in Euclidean distance, as shown in Figure 2 (bottom right). Therefore, since the Neural ODE fits any dynamical system tangentially in time, it is important to observe dynamics sufficiently long enough to differentiate between the models. At very long intervals, the distance starts to decrease as all trajectories converge to an attractor based at the origin, which is special for the spiral dynamical system and not present in the neural data. In summary, the results on the synthetic spiral data show that it is possible to use this method as a system identification, but the systems need to be simulated for a long enough time interval for the differences to manifest, as the distances close to the initial condition are harder to tell apart, since the output of the LSTM minimizes the prediction error at the first timestep.

### 3.2. Fitting differently parameterized firing rate models to resting state fMRI data

The dynamics of BNMs were influenced by many parameters and were tuned to fit fMRI data. Therefore, it was essential to test whether this method allowed us to parameterize different BNMs. The FRM was chosen as it has been well studied in the past, and the Neural ODE can be validated by reproducing previous estimates (Cabral et al., 2012).

**Figure 3:**
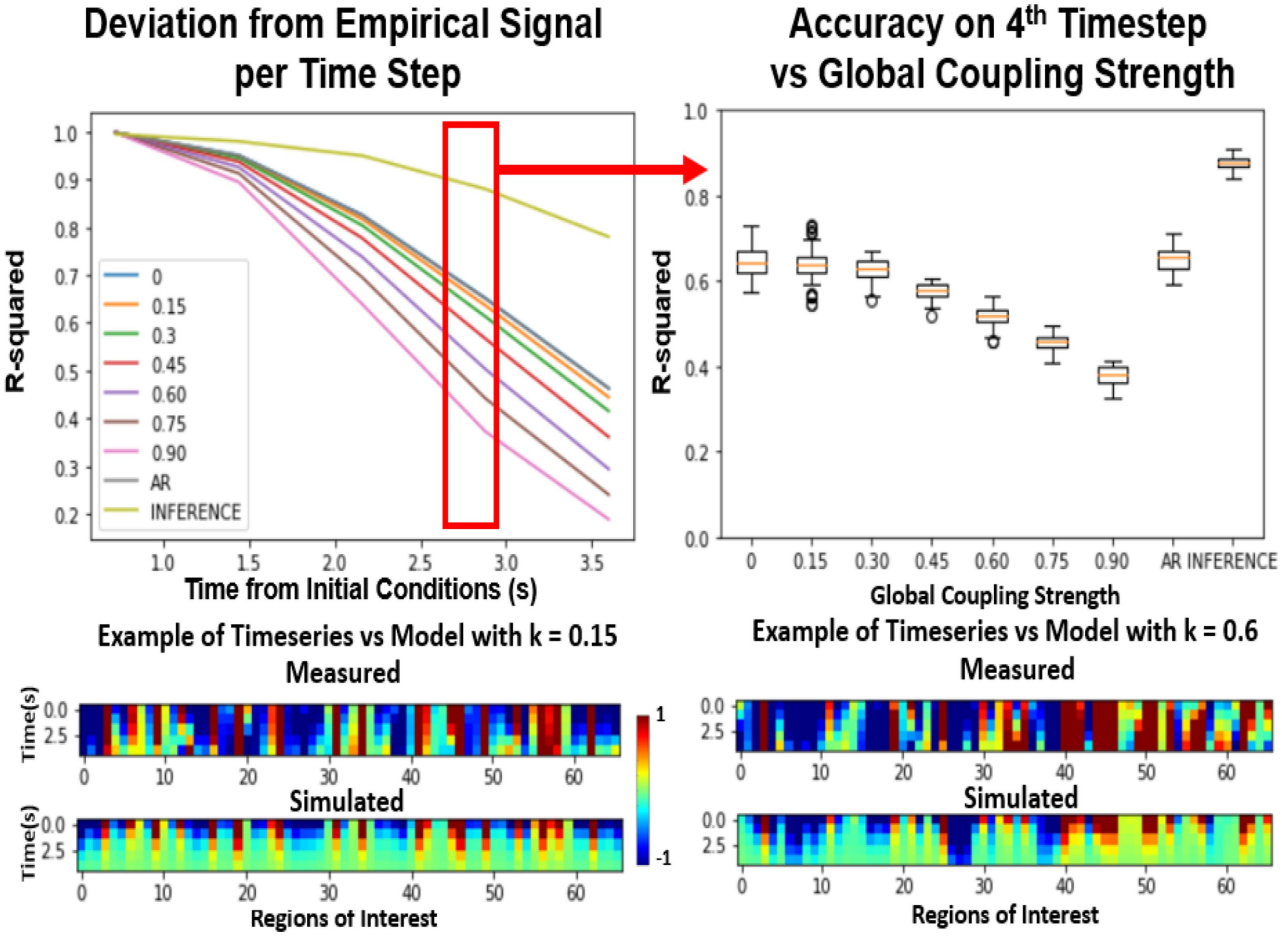
Effects of global coupling in the noiseless firing rate model. Evaluating the FRM (Eq. 1) with different global coupling parameters. The performance was quantified by the r-squared value between the simulation and the model. Top Left: Error per timestep from the estimated initial conditions for various different parameters compared. Examples of the model timeseries vs. the resting state timeseries are given on the bottom at two different parameterizations but the distribution shown in the top panels quantify their performance across 2500 trials across unseen test data. Top Right: At the fourth timestep, the distribution of the r-squared across all the models is plotted. For the FRMs, the accuracy decreases with the increase of global coupling, and the model with zero global coupling performs the best. The Autoregressive (AR) model utilizes the LSTM for the first timestep prediction and then outputs the next prediction as the previous timestep and does as well as the FRMs with zero global coupling. The inference model using LSTM at every timestep as implemented in Kashyap and Keilholz (2020) performs the best in terms of accuracy, but the model dynamics are unknown as they are implemented using deep learning. (Mars, 2020).

Figure 3 compares various differently parameterized FRM models and three Machine Learning Null models in terms of their ability to reproduce the future trajectory. After estimating the initial conditions, the distribution of distances between the predicted and the actual trajectory was plotted over time. At the first timestep (Figure 3, top left), all the models perform relatively similarly as a direct result of minimizing the loss function using an LSTM. Similar to the spiral example, the models diverge in performance when moving forward in time. Surprisingly, at the fourth timestep (Figure 3 top right), the exponentially decaying null models with a zero global coupling, and the autoregressive null model without a BNM (labeled as AR) perform better than models that contain the brain structure. This suggests that introducing any BNM, decreases the accuracy of the model, and the model performs best using just the LSTM predictions. The null model utilizing LSTM inference (Kashyap and Keilholz, 2020) performs the best and represents an estimate of an upper bound in predictability of the rs-fMRI signal.

**Figure 4:**
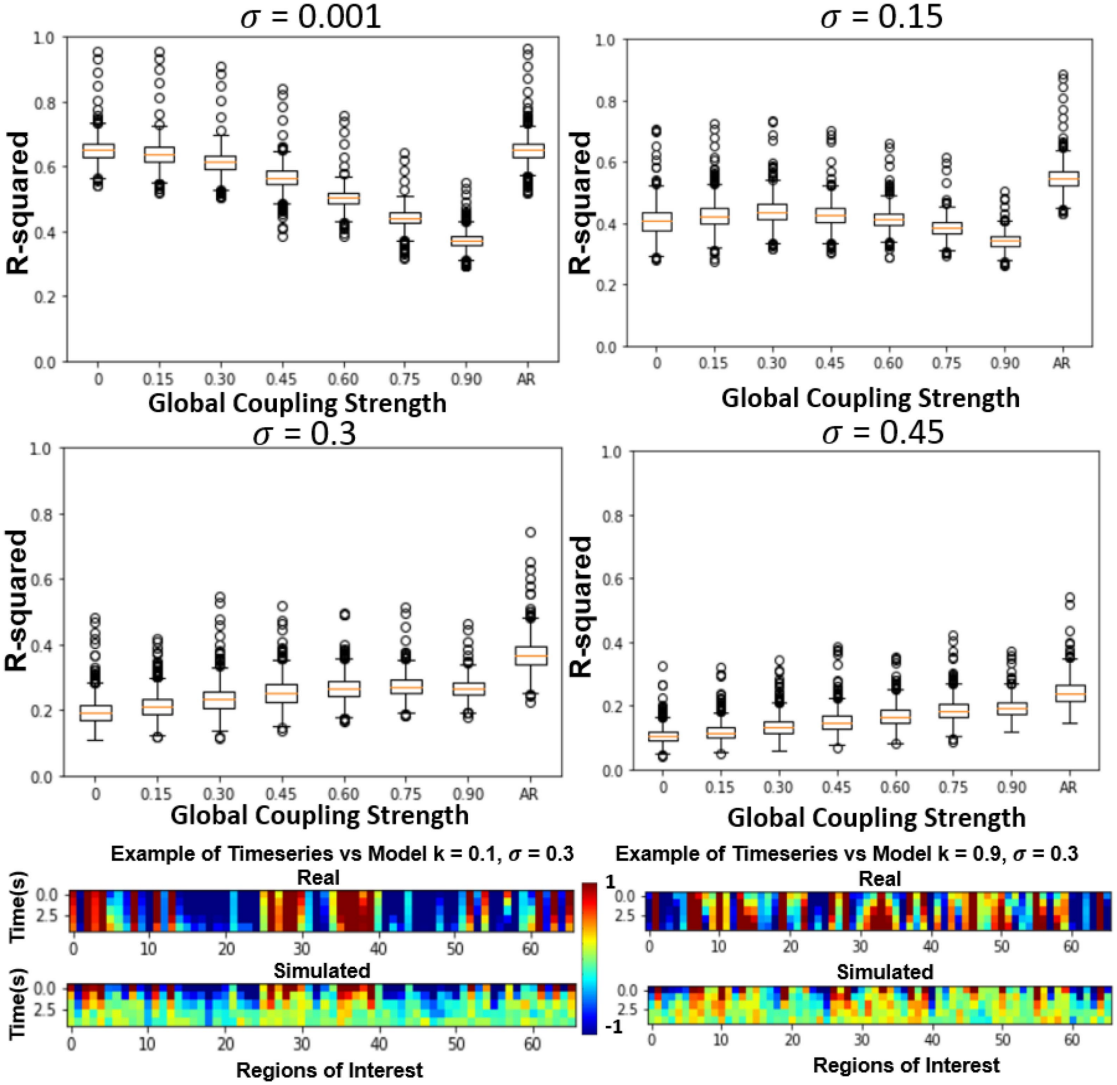
Effect of varying global coupling and noise on the 4th timestep accuracy of the firing rate model. The effect of varying the two parameters in the FRM, the global coupling K and the standard deviation of the noise σ. The introduction of noise lowers the accuracy of all models but does so in an uneven fashion. At low levels of noise, the exponential only model (*k* = 0) outperforms the BNMs with structural connectivity matrices. However, with increasing noise power, the BNM with stronger global connectivity matrices appears to outperform the naïve exponential models. The autoregressive model’s performance also worsens with increasing noise levels, although it still outperforms the FRMs regardless of the coupling strength, the gap between them is reduced. (Mars, 2020).

However, interestingly this trend completely reversed in the presence of noise. In Figure 4, both the standard deviation of the noise as well as the global coupling parameter have been varied. The models with low global coupling perform better at low noise levels, but as the noise level increases, the BNMs with stronger network effects outperform those with low levels of global coupling. This suggests that noise plays a critical role in establishing the parameters of the FRM, and the properties of the structural network become more significant when the system has high noise. The overall r-squared of the models decreases with the introduction of noise, but the rate at which they diverge from the measured trajectories appears to depend on the global coupling parameters. Previous FRMs that used the same brain parcellation, were simulated with *k* = 0.9 and *σ* = 0.3, and noise was seen as essential in simulating the BNMs (Cabral et al., 2012). However, since both parameters are unknown and the overall r-squared decreases with the introduction of noise, just based on varying these two parameters it is difficult to conclude which parameterization yields the best result using this approach. Moreover, the AR null model still performs better than the introduction of a BNM, but the difference is much smaller than before. The inference model is not included in the noise estimations, as the dynamical system is represented as a RNN and cannot be manipulated in a controlled manner as the other models.

**Figure 5:**
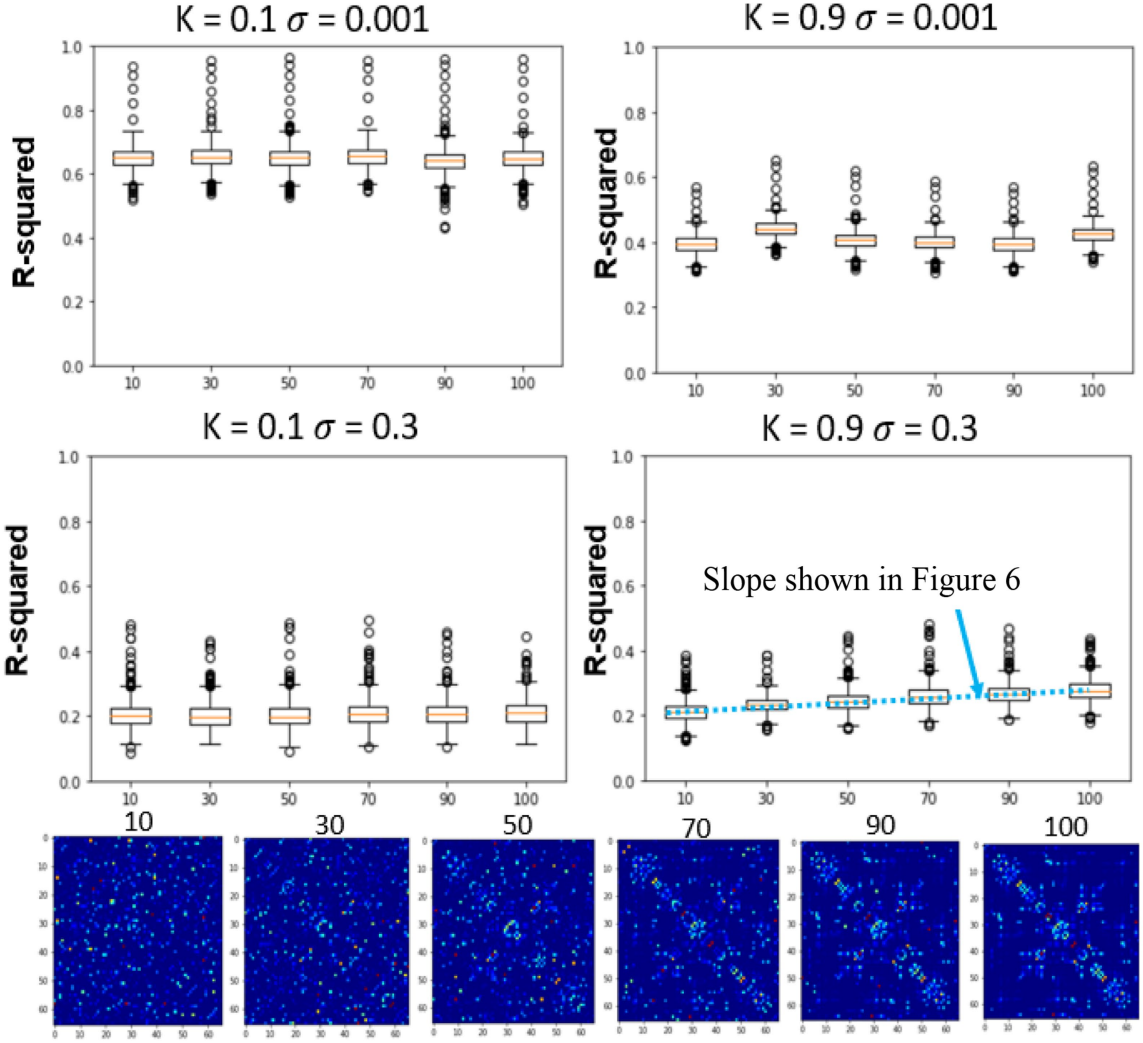
Effects of global coupling, noise, and structural connectivity on the 4th timestep accuracy of the firing rate model. Examining the effects of parameterization and estimating the correct SC. At top left, we show the results of changing the structural connectivity for a low global coupling model and low noise levels. It does not vary as a function of the structure and performs relatively similarly to the LSTM only null model. At high global coupling and high noise levels, the models show that they are more of a function of the correct structural network (bottom right). The slope across the performance of different structural connectivity (bottom right of Figure 5) is used as a metric in the next section to solve for global coupling and noise levels. (Mars, 2020).

The introduction of noise changes the resulting dynamics, which is apparent from the time traces. Illustrated in bottom of Figure 4, the trajectories without noise decay to zero, in line with well-known analytical solutions to the consensus equation, where the values of a connected network with eigenvalues less than 1 converge to the origin (Mesbahi and Egerstedt, 2010). However, the introduction of noise results in more complex trajectories as depicted in Figure 5 bottom, where the values do not decay to zero, but rather randomly oscillate around the origin which serves as an attractor in the system (Cabral et al., 2011). The role of the structural network, in this case becomes more important as it integrates the noise inputs through the network, and results in trajectories more similar to the measured rs-fMRI signal.

### 3.3. Differentiating between BNM due to differences in structural connectivity

In the previous section, only the parameters of the FRM, the global coupling strength as well as the magnitude of the noise were changed. However, in this section, the effects of simulating six different SC matrices at high and low different global coupling (*k* = 0.1, 0.9), are quantified while varying the high and low noise levels (*σ* = 0.001, *σ* = 0.3). The SC matrices are varied from the measured SC by flipping edges and results in SC seen in Figure 5 bottom row. The r-squared value at the fourth timestep, between the different models is plotted in Figure 5 top two rows. Unlike the previous sections where the correct parameter values were unknown, in this experiment, the original SC is expected to outperform the models with altered SC configurations.

At low noise levels (*σ* = 0.001), there was no significant relationship between altering the structural connectivity and either of the coupling strengths. However, at the high noise levels (*σ* = 0.3), although the model has a lower r-squared than at the low noise levels, the effects of the network are evident, with the original SC configuration outperforming the corrupted SC configurations for both low and high coupling strengths. The trend was once again more prominent for the high global coupling (*k* = 0.9) than the low global coupling (*k* = 0.1).

### 3.4. Estimating the parameterization and noise level of the firing rate model

**Figure 6:**
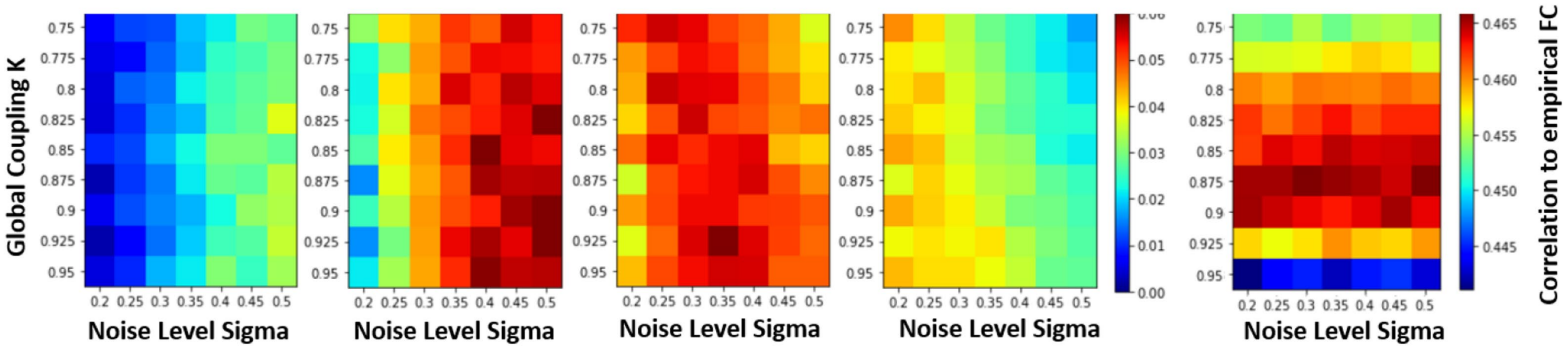
Parametrization of FRM using short term measures vs. parameterization using long term measures. Examining the effects of changing the structural connectivity matrices vs. accuracy (slope in Figure 6) under different parameterization/noise levels of the Firing Rate Model. The sensitivity of the system to changes in the SC matrix is the only metric where the expected outcome is observed, where the original structural matrix outperforms a random one. The change in r-squared value is represented using a color bar and is plotted for different timesteps. At the fourth timestep (*t* = 2.88) the maximum differences occurs on all the models regardless of the parameters, since at that time step the system has diverged enough from the initial conditions and while not far enough in time to cause the overall r-squared to drop too low and make the models indistinguishable. At this timestep, a maximum slope occurs at (*k* = 0.925, *σ* = 0.35). Comparison to the traditional parameterization is shown on the right. Here the differently parameterized FRM models were simulated for 20 min and then the FC matrices of the resulting simulation were compared against the empirical FC. The traditional approach has a maximum at [0.875, 0.3], and previous reproductions of this experiment found a maximum at [0.9, 0.3] (Cabral et al., 2012), showing good agreement on which BNM recapitulates rs-fMRI in both short and long term measures. (Mars, 2020).

In the previous sections, the effects of varying the global coupling and noise levels on the accuracy of the simulated system were explored, but the relationship between the system and the underlying structural connectivity remained unclear. To investigate this, the system was simulated with different SC matrices while varying the noise levels and global coupling values. The slope was calculated at different time steps to find where the system was most sensitive to the underlying structural connectivity to find the correct parameterization of the models. The slope is plotted from timesteps 2 to 5, across different global coupling values and noise steps in Figure 6. At timesteps close to the initial condition, higher levels of noise are needed to differentiate the systems sensitivity to the structural matrix. However, at later timesteps, the opposite is true where higher noise levels perturbs the system too much and the overall r-squared drops so low that the models become indistinguishable to each other. Therefore, the fourth timestep is used where the max differentiation between models occurs regardless of the coupling values, where the trajectory is far enough from the effects of the LSTM fitting and close enough in time to test the predictability of the models. At this timestep, the maximum occurs at the values (*k* = 0.925, *σ* = 0.35). This value is very close to what has been used to be simulate FRMs (*k* = 0.9, *σ* = 0.3) from previous publications (Cabral et al., 2012). Their approach of parameterization here has been reproduced in Figure 6 (right most panel), which calculates the FC of a 20 min simulation and correlates the FC with the FC of the empirical data. The maximum at [0.875, 0.3] is in good agreement with the short-term measures and the previous estimates.

### 3.5. Evaluating the initial conditions of the NODE model

**Figure 7:**
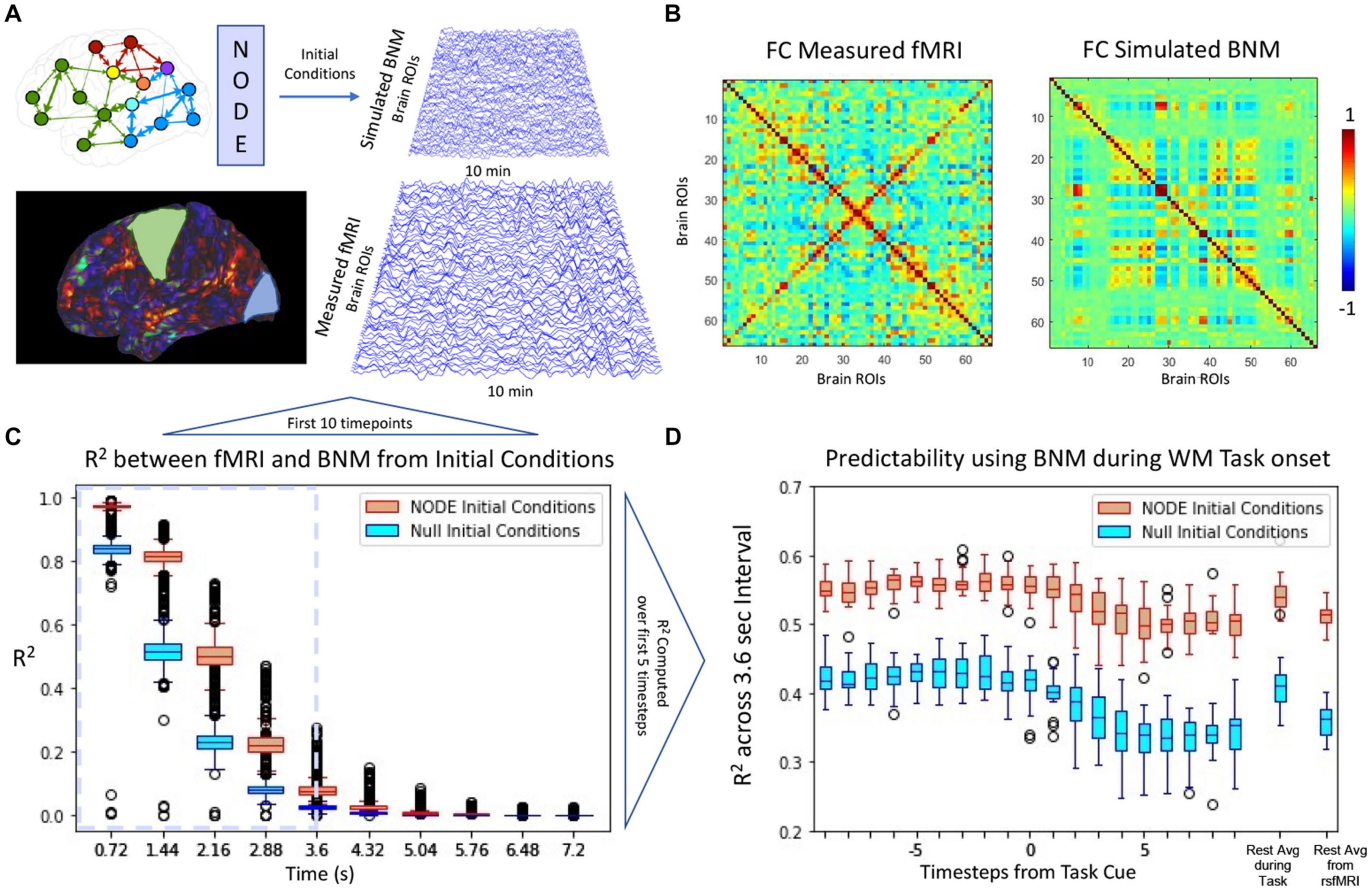
**(A)** Comparison of BNM simulation with measured brain activity derived from rsfMRI. Simulation of 66 regions for 10 min is first synchronized using the NODE algorithm such that the initial conditions are set for the BNM. **(B)** Functional connectivity of the simulated model and the empirical signal (cross-correlation ~0.45). **(C)** For the first few timepoints, the simulated signal follows trajectory more closely using the solved for initial conditions than using the measurement at that timepoint as initial conditions (null initial conditions). The signals diverge due to the noise added at the simulation as well as the drift that occurs in rsfMRI. We define the Region of Predictability (RP) based on the first 3.6 s and compute the across all timepoints in that interval, at which the NODE initial conditions perform significantly better than the null model. **(D)** The RP is plotted from every timepoint during a working memory task, where the cue is presented at 0. All models lose predictability during the task onset and predictability stays lower during the task interval compared to the average rest average. The predictability during the task onset is closer to the predictability during the rsfMRI scans than during the resting state portion of the task scans. (Mars, 2020).

In the previous sections, the application of NODE algorithm to correctly bias the BNM models and recover coefficients by using short term metrics that match those of previous literature were highlighted. In the following section the NODE (*k* = 0.925, *σ* = 0.35) is utlized to evaluate the initial conditions of the algorithm vs. null initial conditions. The null initial conditions were generated by taking the previous timestep, and integrating the BNM from that timestep. For long term simulations shown in Figure 7A, the functional connectivity is characterized in Figure 7B with a correlation of 0.45 with the empirical measured signal. While the initial conditions did not change the functional connectivity of a long term simulation, Figure 7C illustrated that the trajectories from NODE initial conditions followed the signal more closely than the null initial conditions. Moreover, this difference is also present when comparing rest vs. task as shown in Figure 7D, indicating that the algorithm is producing non-trivial results for its initial condition prediction.

## 4. Discussion

### 4.1. Overall discussion and significance

The study proposed the use of the Neural ODE technique for estimate initial conditions in different candidate BNMs and subsequently evaluating the predicted trajectories compared to the real data. To test this methodology, the technique was first applied to a well-studied spiral dataset, which demonstrated its ability to correctly identify parameters in a constructed example of system identification where the ground truth was known. The method was then applied to fit different Firing Rate BNMs to neural fMRI data by varying their parameterizations, noise level, and by changing the structural connectivity. By using all three, the system was correctly able to identify the parameters of the FRM, which were close to previous estimations of the model’s parameterization using the same whole brain parcellation (Cabral et al., 2012). Moreover, these initial conditions were shown to be non-trivial as they perform better than just using the previous timepoint as initial conditions.

Therefore, pertinent information to parameterize BNMs is present in short term trajectories analysis. Unlike older metrics, this technique allows for direct timeseries comparisons between the theoretical models with measured experimental data, and circumvents the reliance on a certain metric/interpretation of rs-fMRI. Therefore, this technique provides a unbiased metric that can be extended to compare and parameterize more complex BNMs. Moreover, it allows pathway forward in studying whole brain dynamics on a faster timescale, and illuminate what our current theoretical models can and cannot explain in terms of transient dynamics.

The interpretation of the initial condition is difficult in rs-fMRI, as rest is not labeled with respect to stimulus, but they can provide information of the phase of cyclical brain processes such as quasi periodic patterns (QPPs) that have been identified in the literature to exist in both rest and task data (Thompson et al., 2014). The phase is thought to be important improving the correlates to response time in task fMRI, and thus estimating these conditions might prove relevant in understand the current state of these cyclical brain processes (Abbas et al., 2020).

### 4.2. Parameterization and noise levels of the firing rate model

The study focused on the Firing Rate Model (FRM), which is the simplest of the BNM used to simulate rs-fMRI. The model has two variable parameters - the magnitude of global coupling and the level of noise (Eq. 1). Previous literature has suggested the global coupling value of a traditional FRM is set slightly less than 1, around 0.9, which is just before the system becomes unstable. The noise is usually set around 0.3 and global coupling to 0.9 for this parcellation scheme (Cabral et al., 2012; Kashyap and Keilholz, 2019), where closer toward zero it simplifies to the well-known consensus problem where the timeseries converge to the attractor at the origin (Mesbahi and Egerstedt, 2010), and at higher degrees of noise the system becomes completely chaotic and non-deterministic. Searching for the correct parameterization of the FRM between the global coupling and the magnitude of the noise is therefore an important to simulate the model in the correct regime.

In practice, this relationship was not so easily ascertained by analyzing the short-term trajectories as it was confounded by the presence/absence of noise. The simulated trajectories that were the closest to the empirical trajectories were models that contained no noise resulting in a parameterization of a trivial exponential decay null models (Figure 3). However, in the presence of noise, the role of network structure became important, as at higher global coupling values the signal would deviate less from the empirical trajectories. The role of the structural connectivity here can be thought to averaging out the noise, and the trajectory became more robust to local deviation due to noise introduced at each ROI. Supporting this argument, Figure 5 demonstrated that in the presence of noise, the models also exhibited a dependence to changes in the structural connectivity, where the true structural connectivity resulted in dynamics closer to the empirical signal than noisy perturbations of the original structural connectivity. Although the exact value of noise cannot be solved by maximizing the r-squared accuracy while varying the noise amplitude as it results in favoring noiseless models; at the right noise/ global coupling parameterization, the accuracy of the FRM should be maximally dependent on the correct structural connectivity. This hypothesis was tested in Figure 6, where the change in accuracy of due to the changes in the structural connectivity was plotted. Using this metric, the FRM with (*k* = 0.925, *σ* = 0.35) is the most dependent to changes in the structural connectivity and is very close to previously known values that was being used for the FRM (*k* = 0.9, *σ* = 0.3) as well as our computed maximum using long term FC estimates (*k* = 0.875, *σ* = 0.3) (Cabral et al., 2012). The previous process used long term FC as a metric to maximize to parameterize the models, rather than using the short trajectories as in this manuscript, but here they show they give similar estimates on which FRM is closest to measured rs-fMRI dynamics. The evidence that these two values are close, suggests that our approximation of the true underlying dynamical system is at least scale free across the observed timeframes and models that have been used to simulate long periods of time, can capture meaningful dynamics in the shorter timeframe.

### 4.3. Comparison to other neural ODE architectures

The original Neural ODE implementation uses a backward time architecture, where the timeseries is inverted and fed into the RNN network, such that the first timepoint is fed into the RNN last and the final prediction is used to infer the initial condition of the whole timeseries and then integrated forward in order to compute the loss function (Chen et al., 2019). They do not evaluate the RNN prediction at every timepoint like in our implementation, but explicitly state that such an architecture would speed the training process. The Tensorflow RNN implementation page also recommended a parallel use of the RNN in order to speed up the training process.^1^ The innovative backwards time architectural method gets rid of the initialization problem of the RNN that exists in our forward time implementation but runs into a causality problem where future inputs influence the predictions of previous initial condition. Because BNMs are defined as a function of previous network activity, and because our intended use of the trained model is a continuous correction of the accompanying BNM model, the time forward architecture is used in order to solve for the initial conditions. The other significant difference is that our implementation of the Neural ODE also uses a LSTM after the ODE integration (Chen et al., 2019). This methodology is extensively evaluated in Kashyap and Keilholz (2020) but confounds our goal of comparing the fit of different dynamic systems, so it is simply presented as a null model labeled as inference in this paper. This model outperforms all other models in terms of short term prediction, but cannot be manipulated as in terms of the noise level, coupling strength or other meaningful biological variables. Rather it represents an estimate of an upper bound in terms of predictability seen in the rs-fMRI dataset.

### 4.4. Comparison to other techniques in literature

The Neural ODE algorithm presented here is a relatively new technique first presented in 2019. To our knowledge this exact technique has not been applied in the context of fitting whole brain models with empirical rs-fMRI data. However, our methodology is quite similar to our own previously published work (Kashyap and Keilholz, 2020), but differs in the important following manners. In the previous paper, the system was trained in a very similar manner, but in the generation of new data from the initial conditions the older methods utilized the entire Machine Learning architecture, LSTM and the Brain Network Model to synthesize new data, whereas in this paper, the future timeseries is generated from initial conditions by integrating the Brain Network Model. The older method allowed to generate more realistic brain data and replicate brain dynamics better than traditional BNM as it utilized the LSTM in every timestep. However, this brought into unknowns into the dynamics and it was not possible to evaluate the BNM on their own. Therefore, in order to isolate the performance of BNM for the purposes of system identification, the LSTM was excluded from the inference process and was only used to generate initial conditions. A recent preprint (Wun, 2020) also utilizes the Neural ODE approach to fit to rs-fMRI data. However, in that methodology it does not use the Neural ODE tool to fit trajectories from the BNM rather analyzes latent variables of a model to predict task states. Many other approaches have started using different techniques for uncovering principles of dynamical systems in order to represent rs-fMRI (Zalesky et al., 2014; Hjelm et al., 2018; Vidaurre et al., 2018; Nozari et al., 2020; Singh et al., 2021). Nozari et al. (2020) uses a similar r-squared metric to quantify the difference at the first time point prediction but does not extend this by predicting further out in time. In this paper, to our knowledge is the first to use these tools for comparing short term trajectories of given BNMs to measured rs-fMRI data.

### 4.5. Assumptions and limitations

The error from the model’s prediction comes from multiple different sources such as (1) the mismatch between the differential equations and the actual dynamics, (2) from the error in predicting the initial conditions, and (3) inadequate descriptions of structural connectivity and/or the lack of including subcortical areas in the simulations.

1. A major limitation of this approach is to have an estimation of the underlying dynamical system that represents the data. This requires vast knowledge of what model including the specific parameterization might fit the dataset. However, the Neural ODE system is able to tangentially fit any dynamical system even trivial ones such as the exponential decay. Therefore, it is not really necessary to have a really good estimation of the underlying system and can be tested how well they predict subsequent timesteps.
2. We assume that for any assumed dynamical system the error from the RNN is uniform no matter what the function is, and the subsequent error calculated from the trajectories is due to the mismatch between the data and the dynamical system. However, this might not be true, and more complex models might have a larger errors in estimating initial conditions and therefore is a potential confounder in our analysis.
3. The inadequate description of the brain network is also a limitation and can be improved with higher resolution parcellations and subcortical areas. Another major drawback is the network is quite ill-defined because there is no consensus what constitutes a ‘cohesive’ neural population. However, different atlas and network definitions seem to give similar results suggesting that the principles of BNM are at least consistent across many parcellation schemes that are used today. However, results from previous literature, show that a more detailed description of the network only improves the models performance and its ability to recapitulate rs-fMRI and that coarser models are good enough for a proof of concept application.

Moreover, another major assumption and limitation of the approach is our choice of metric, r-squared used to compare the distance between two high dimensional vectors. It assumes that better models have a higher r-squared value, although they might be explaining trivial components of the signal. Other metrics such as derivative, or the relative phase between different regions of interest might prove as a much more useful metric to compare the predictions against the empirical signal. The method also introduces another variable on when to evaluate the differences of the model. Close to the initial conditions the trajectories are too close to differentiate, and as seen from the null models where the output of the LSTM already captures a large amount of the variance in the signal. Too far from the initial conditions yields trajectories that are too far away from empirical measurements and all models become completely indistinguishable. For our results, the fourth timestep (2.88 s) was the most useful in differentiating between models, but this could vary from implementation and careful consideration needs to be used in interpreting the results and is a limitation in the approach.

### 4.6. Future applications

The Neural ODE techniques has a lot of potential as an additional tool in conjunction with BNM. It can be used to evaluate any differential for brain data in real time by solving for the initial conditions. Moreover, it can be used to compare across increasingly disparate brain models that are being constructed for specific applications. For individual data, it seems especially promising, since the trained network can make predictions on an individual fMRI data and thus parameters of the BNM as well as the structural connectivity can be adjusted on the individual level. Furthermore, it allows for modeling BNM trajectories during task fMRI, where the component of the signal due to network activity can be estimated, and enhance the response due to stimulus. Our results in Figure 7, indicate that the initial conditions of the Neural ODE outperform the null estimation of using the measurement as initial condition, and therefore results in trajectories that better recapitulate the short term trajectories. Therefore in the future, this algorithm can aid in separating network and task dependent activity intrinsic to fMRI.

For future approaches on more complex BNMs, it might be easier to assume the noise level and then the parameters can be solved in a more straightforward manner. The noise level seems to be endemic in the system, and rather than a parameter of the model. Since our mean surface area parcel is 858 mm^2^ according to our atlas, we estimate the cortical noise per area to be $N\left(\;\mu =0,\sigma =0.35*\mathrm{Surface}\;\mathrm{Area}/858\;mm^{2}\right)$ and not to be a function of BNM. Once the noise level has been established, the structural perturbations are not necessary and the coefficients of the BNM can be determined directly by comparing the r-squared of the models with the empirical signal. In this manner many more complex BNMs can be compared against each other.

## 5. Conclusion

This manuscript investigated whether by solving for the initial conditions of a Brain Network Model for a given observation of rs-fMRI data using Neural ODE, the estimated BNM trajectories based on these initial conditions would serve as a metric to differentiate between BNMs and the measured rs-fMRI timeseries. The approach used several different FRM to fit to the rs-fMRI data by varying the global coupling, noise, and structural connectivity. The results show that the parameterization of global coupling and noise that maximizes the model’s sensitivity to the structural connectivity, yields a model comparable to earlier parameterizations of the FRM. Therefore, the Neural ODE tool has the potential to differentiate and develop more complex BNMs to fit rs-fMRI data and a path to train the parameters on individual fMRI data.

## Data availability statement

Publicly available datasets were analyzed in this study. This data can be found at: Human Connectome Project.

## Ethics statement

Written informed consent was obtained from the individual(s) for the publication of any potentially identifiable images or data included in this article.

## Author contributions

AK: code, design, and manuscript draft. SP, PR, and SK: design, editing, and advising. All authors contributed to the article and approved the submitted version.

## Funding

This work was supported by H2020 Research and Innovation Action Grant Human Brain Project SGA2 785907 (PR), H2020 Research and Innovation Action Grant Human Brain Project SGA3 945539 (PR), H2020 Research and Innovation Action Grant Interactive Computing E-Infrastructure for the Human Brain Project ICEI 800858 (PR), H2020 Research and Innovation Action Grant EOSC VirtualBrainCloud 826421 (PR), H2020 Research and Innovation Action Grant AISN 101057655 (PR), H2020 Research Infrastructures Grant EBRAINS-PREP 101079717 (PR), H2020 European Innovation Council PHRASE 101058240 (PR), H2020 Research Infrastructures Grant EBRAIN-Health 101058516 (PR), H2020 European Research Council Grant ERC BrainModes 683049 (PR), JPND ERA PerMed PatternCog 2522FSB904 (PR), Berlin Institute of Health & Foundation Charité (PR), Johanna Quandt Excellence Initiative (PR), German Research Foundation SFB 1436 (project ID 425899996) (PR), German Research Foundation SFB 1315 (project ID 327654276) (PR), German Research Foundation SFB 936 (project ID 178316478) (PR), German Research Foundation SFB-TRR 295 (project ID 424778381) (PR), and German Research Foundation SPP Computational Connectomics RI 2073/6-1, RI 2073/10-2, and RI 2073/9-1 (PR).

## Conflict of interest

The authors declare that the research was conducted in the absence of any commercial or financial relationships that could be construed as a potential conflict of interest.

## Publisher’s note

All claims expressed in this article are solely those of the authors and do not necessarily represent those of their affiliated organizations, or those of the publisher, the editors and the reviewers. Any product that may be evaluated in this article, or claim that may be made by its manufacturer, is not guaranteed or endorsed by the publisher.

## Footnotes

^1^ tensorflow.org/guide/keras/rnn
