# Supplementary material for: A deep learning approach to estimating initial conditions of Brain Network Models in reference to measured fMRI data
Source: Front Neurosci. 2023 Jul 17;17:1159914. doi: 10.3389/fnins.2023.1159914 (PMC10390027; doi:10.3389/fnins.2023.1159914)
Supplement: Supplementary file 1 [file Data_Sheet_1.pdf]

1. Supplemental Material

1.1 Neural ODE algorithm

The following steps outline the sequence of execution used to train the system.

1. The input data is shown in the bottom left corner Figure 1, where it consists of sequence of timeseries $x_0, x_1, x_2\ldots$ to x_t. They are fed into the LSTM network one at a time. The Tensorflow LSTM implementation of the RNN remembers up to x_{t-N} values, where N is the length of the LSTM. N is set sufficiently large such that the performance of the LSTMs converges to its optimal value in M timesteps where $N>M$ (see supplementary section 7.3).
2. For the current input x_t and the LSTM with its hidden state p_{t-1}, the LSTM estimates the initial conditions as a normal distribution with a mean and standard deviation (μ_t,σ_t). We then sample an estimate of the initial condition h_t from this distribution. This is not explicitly shown in Figure 3.1 but is used to avoid overfitting.
3. Calculate the loss function at point t (Figure 3.1 bottom right): Use a 4th order Runge-Kutta solver to compute $ODESolve(f,h_t)$, where ODEsolve represents the differential equation solver to integrate over the interval t to $t+1$. This point is assumed to be close to the next measured datapoint x_{t+1}. The Euclidean Distance between these points is used as the loss function. A graphic illustration of this distance is shown in Figure 3.1 bottom right.
4. The gradient is then calculated by averaging the loss function across all timepoints and across all batches, and is backpropagated through the Neural ODE using Tensorflow in order to train the network.

The algorithm trains until the loss function distance approaches zero. In practice, this is implemented by training to L number of steps and L is varied to make sure that the algorithm produces consistent results (supplementary section 7.4). The Neural ODE algorithm assumes that the ground truth distance between the predicted initial conditions and the true initial conditions also converges to zero when the loss function distance is minimized. The algorithm is initialized with a null hidden state, and slowly updates its hidden state as it sees more observations over time.

1.2 TensorFlow Implementation

Supplementary Figure 1 Neural ODE Architecture

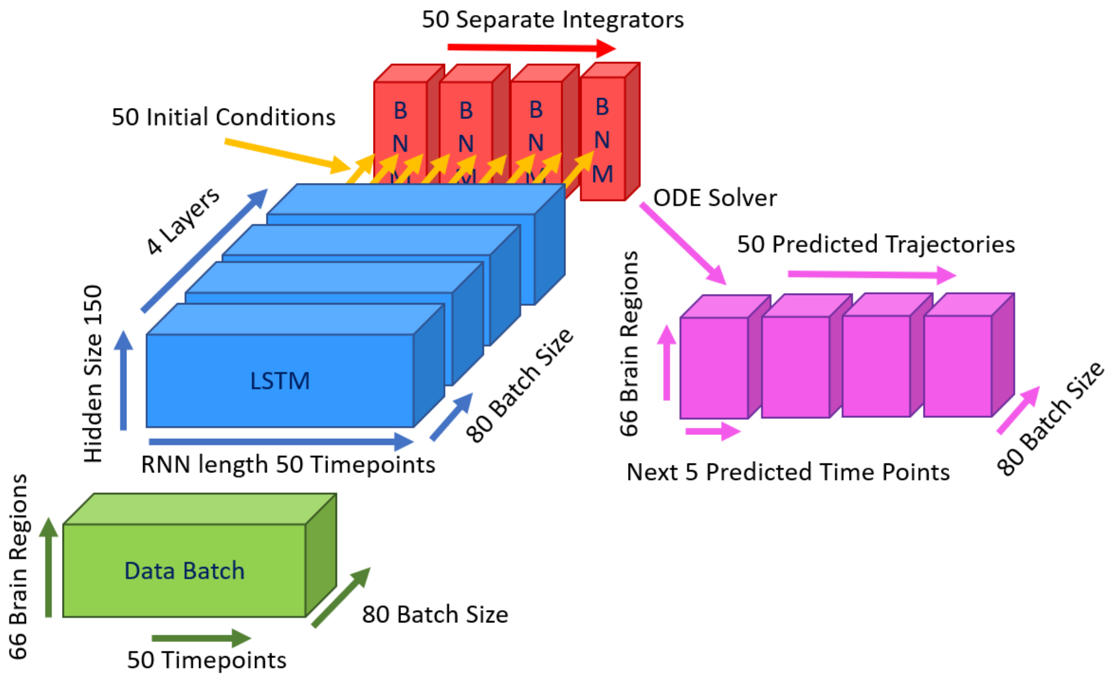

Neural ODE architecture as represented by tensors with their appropriate sizes. The Data Batch consists of 50 consecutive timepoints from 66 Brain Regions of 80 subjects (batch size). The batch is fed into a 4-layer LSTM network, with a hidden size of 150. The initial conditions of the LSTM are set first to zero, and then to the output of the previous batch that contained the last 50 timepoints. The output of the LSTM is transformed via a feedforward layer to represent the mean and standard deviation of the initial conditions. An initial condition for each timepoint is then sampled and fed into the BNM in parallel and integrated by 50 separate integrators for the next 5 consecutive timepoints. The prediction at the next timestep is used to train the system, while the other 4 timepoints are evaluated during testing.

The schematic above is implemented in the architecture shown in the Figure 2, which utilizes parallelization to efficiently train with large amounts of data. The network trains simultaneously using multiple subjects (batching of 80 subjects) and multiple time steps at the same time (50 consecutive time steps in Supplementary Figure 1 but varied in supplementary section 7.4). The input would be a vector from the activity of all brain regions (66 ROIs at one time point) and is 3-dimensional tensor (66 ROI regions, 80 batches, 50 timepoints) and hidden state vector calculated from the previous time step by the LSTM. The LSTM is implemented using the CUDA library for NVIDIA GPUs and the architecture is a 4-layer hierarchical LSTM network. The LSTM is first initialized with a hidden state of all zeros and computes the data sequentially starting with the first timepoint of the 50 timepoints. The hidden state of the first input timepoint is then passed to the next timepoint, and the last state is passed as an input to the next time segment. The hidden state represents the feedback arm of the LSTM and is set to 150 (varied in supplementary section 7.4) and is 4 layers deep. The final output of the LSTM is then transformed from the size of the LSTM (150) using a feed forward Neural Net to the size of the initial conditions before it is fed to the BNM. The initial conditions are represented as a normal distribution with a (μ,σ) and the initial

conditions is sampled from that distribution. The final output represents the mean firing rate of the neural population at that timestep for the Firing Rate Brain Network model (66 for the different brain regions). The model is then integrated for a time interval using a 4th order Runge Kutta ODE solver using the Firing Rate BNM and produces an output representing the activity of the measured signal at the next time signal. The gradient is then subsequently calculated by taking the difference of the predicted output and the next measured timestep and then used to adjust the weights of the neural networks such that the sampled output of the LSTM converges to the initial conditions of the BNM. Since the accuracy is a function of the size of the network with respect to the data size, the parameters for the neural network were determined via sweeping each of these parameters across several values. The details of this process are outlined in supplementary section 7.6.

During testing, the trained LSTM model is used in the inference mode to estimate the initial conditions at each timepoint; the gradient is not computed at this stage. From each of these initial conditions x_{init} estimated from data observation $x(t)$ future timepoints $x(t+1), x(t+2), x(t+3)$ are generated by integrating the according to the equations of the Brain Network Model. In this manner, estimates at the same sampling period as the data are produced and the resulting trajectories are compared with the measured trajectories. To quantify the distance between them, the adjusted r-squared is taken at each future timestep between the vector representing the simulated activity of all 66 regions and the measured mean fMRI activity in the corresponding regions.

1.3 Simulated Spiral Data

In order to validate our approach, we tested our algorithm/architecture that we use on fMRI data, on a toy spiral data set. This spiral data has been used in the original Neural ODE paper (Chen et al., 2018), as well as in subsequent papers in order to test the validity of solving for the underlying dynamical system from noisy data. Our approach is to establish that our algorithm can reproduce the performance of the Neural ODE algorithm (Chen et al., 2018) on the spiral data and this therefore justifies its use on fMRI data. In Figure 3.3, we show how we generate our spiral dataset, namely by integrating a set of coupled differential equations with two state variables (equation shown in the left panel). The phase portrait of the dynamical system is shown, where all the trajectories from any initial condition spiral inwards towards the origin (Figure 3.3 middle). Time is not shown in the graph but is implied, where the first timepoint is on the edge of the spiral and the last one is the one closest to the origin. The derivative is large on the outside of the spiral and then decreases as it approaches the origin. Gaussian noise is added to the integrated trajectory to simulate measurement noise set at sigma = 0.03 (Figure 3.3 right). The goal of the Neural ODE algorithm is to estimate the underlying trajectory from the noisy observations that generated the data. For our experiments, we use the trajectories shown in the middle panel of Figure 3.3 as the ground truth and take the Euclidean distance between this trajectory and the predicted initial conditions as a measure on how well our algorithm performs.

Supplementary Figure 2 Generation of Spiral Dummy Data

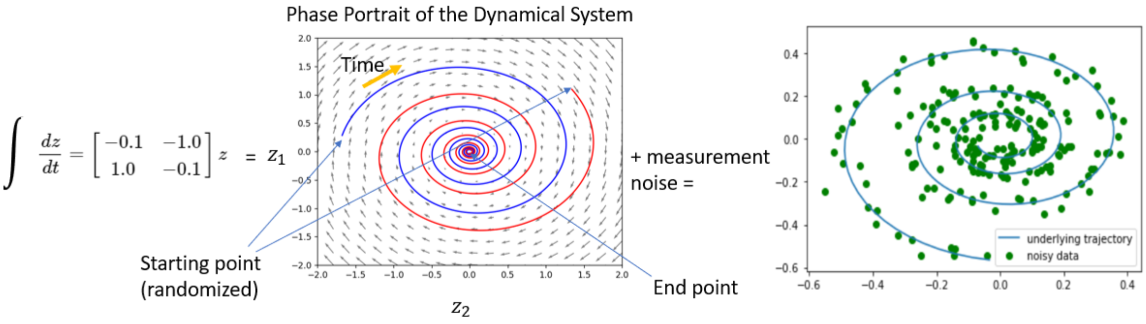

The equation on the far left is integrated and produces the dynamical system shown in the phase portrait (middle). From two random initial conditions the red and blue trajectories spiral towards the origin, getting slower and slower as they approach the origin. The arrows indicate the magnitude of the gradient. We generated 1000 spirals from different initial conditions and added noise to simulate a noisy measurement. This results in the plot on the far right where the green dots represent the data that is fed into the algorithm. The goal of the algorithm is to be able to estimate the underlying trajectory shown in blue (far right) from the noisy observations. This will serve as our ground truth to check our predictions against.

1.4 Validation of the Neural ODE Algorithm on Spiral Data

The spiral data allowed us to test how well our algorithm estimates the initial conditions, in a simplified situation where the initial conditions are known. In Figure 3.4, we show that the predictions of a trained network converge towards the ground truth of the initial conditions for the sample spiral dataset. Starting from the first observation, at the bottom right corner of the spiral, the LSTM makes a prediction based on all the observations it has seen so far, and then a trajectory is produced via integration of the dynamical system of the spiral dataset. The trajectories after seeing 7, 9, and 11 datapoints are shown on the spiral itself. The distance between ground truth initial conditions is also quantified to the right as a function of number of points the LSTM observes. The accuracy during the first couple timesteps is low because the hidden state of the LSTM is not initialized properly, but after enough datapoints are included, it is able to predict the initial conditions within a reasonable margin of error.

Supplementary Figure 3 Infer Initial condition for each Timestep on Spiral Data

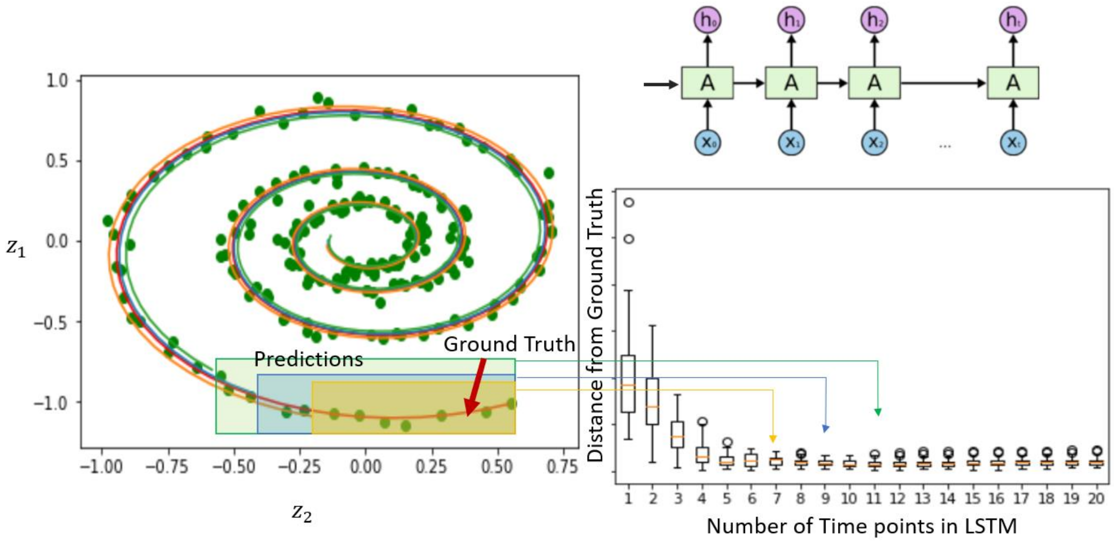

Estimating the initial conditions for the spiral data. The figure on the left shows an example of a trained network estimating the initial conditions using 7, 9, and 11 timepoints. Each timepoint is fed into the LSTM sequentially (shown top right) one datapoint at a time and outputs the initial condition for that timepoint. The trajectories are then integrated and then compared with the ground truth (shown in red). The Euclidean distance between the predicted and the true trajectory is shown in right for a distribution of 40 unseen spirals. The accuracy converges after the first few timepoints during which the LSTM has not yet been initialized properly. After the LSTM has seen enough examples, the estimates converge slowly towards the true spiral trajectory.

1.5 RNN Parameter Estimations on Spiral Data

Next, we test to see how our ground truth distance changes as a function of network size and parameters (Figure 3.5). For our given architecture, we can vary the number of layers (network depth), the size of the hidden layers, and the length of the LSTM network. The length of the LSTM network is a parameter due to Tensorflow implementation of LSTM and limits the number of previous seen observations. We followed Tensorflow's guidelines and biased it until the error converges during the time period contained in the hidden size of the RNN (in Figure 3.5 after 7 or 8 timepoints where the length of the LSTM was set to 15 previous timepoints). We instead chose to test the effect of the hidden size. The depth of the network was kept to three layers, as the spiral dataset is small, and is varied for the neural dataset. The hidden size represents the feedback arm of the RNN, and larger hidden size allows for more complex relationship with previous data observations. We show in Figure 3.5 bottom right, that larger the hidden size (such as 80) the loss function tends to converge in fewer training epochs. Moreover, after they are trained, these networks are more accurate with fewer datapoints (Figure 3.5, left), and are more sample efficient in extracting information from previous data. There are also observable differences in their accuracy, as illustrated with a sample spiral predicted after each network has observed 10 datapoints. However, the difference in prediction between 20, 40 and 80 sized networks is less pronounced especially after observing many datapoints, suggesting that regardless after many data observations the system converges roughly to the same error. This points to a certain robustness of convergence of the loss function with regard to parameter variations for long time sequences.

Supplementary Figure 4: Effect of Network Size on Initial Condition Predictions

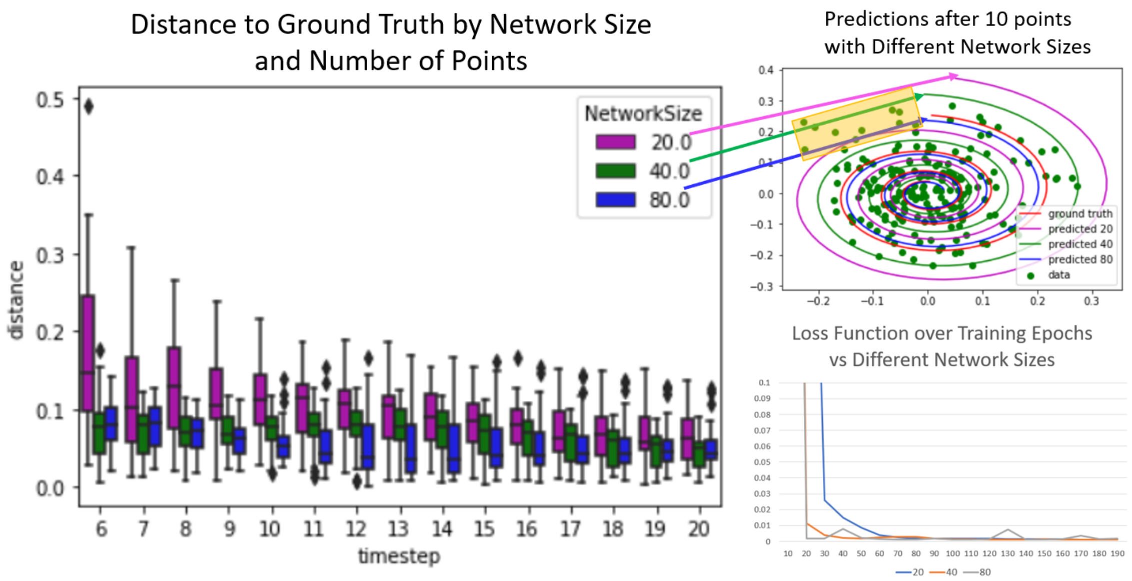

The effect of the network size in its ability to converge to the true initial conditions with fewer samples. Three network sizes of the hidden state (20, 40, and 80) are compared while keeping the depth of the network and the number of batches constant. The plot on the bottom right shows that in all three architectures the loss function approaches zero and plateaus at roughly the same value. However, the larger networks are more sample efficient, meaning that with fewer samples, they are able to estimate better initial conditions. This is shown on the left, where the largest network (80) has the smallest distance to the ground truth. After the LSTM has viewed enough samples the differences between the networks vanishes. An example on a single spiral is shown (top right), where the predictions after seeing 10 datapoints are 0.11, 0.08, and 0.04 apart from the ground truth for the three differently sized networks at 20, 40, and 80 respectively. The actual pink and green data do not cross any of the initial data, because the algorithm only estimates the initial conditions which are not precise for smaller networks, and since we are integrating from the initial conditions, the resulting trajectories are far away from the future datapoints which have not yet been seen by the Machine Learning Network.

1.6 RNN Parameter Estimations on fMRI data

For the neural dataset we have a much larger state space having at least 66 state variables. Therefore, we tested a number of different parameters as listed below in the Table 3.1. For a given BNM, the r-square accuracy was calculated for different parameters of the machine learning network. The base parameters were chosen from Kashyap et. al 2020 with 600 iterations, 50 for length of RNN, 150 for hidden size, and 4 layers and across each row in the table we change one of these parameters at a time, to see how it effects the performance of the algorithm. The accuracy doesn't change a lot when the parameters are varied as shown in Table 3.1 suggesting that the network is maximizing the information transferred from previous states like in the spiral example.

Supplementary Table 1: R-squared Accuracy at the 3rd timestep across different RNN Parameters

**Parameter Evaluated**				
**Number of Iterations**	200	600	800	1000
	0.729 (0.02)	0.743 (0.021)	0.739 (0.022)	0.744(0.02)
**Length of RNN**	30	40	50	60
	0.741(0.03)	0.744 (0.028)	0.743 (0.021)	0.741(0.024)
**Hidden Size**	100	120	150	180
	0.733(0.02)	0.734 (0.02)	0.743 (0.021)	0.741(0.022)
**Number of Layers**	3	4	5	6
	0.738(0.02)	0.743 (0.021)	0.739(0.02)	0.730(0.02)

Evaluation of network parameters of the RNN. The mean r-squared (standard deviation) are given for different sized networks on the Firing Rate Model (k=0.6) on the third timestep prediction for 2500 short resting state fMRI trajectories.

1.7 Session Convergence and Metric Choices on fMRI data

The average loss over 10 epochs each with 50 timepoints is plotted in Figure 3.6 left. The epochs are contiguous over time, where the hidden state of the first epoch is the input for the second epoch. The loss is the mean squared error between the predicted and the next timepoint. The first batch has a large error when the RNN is not properly initialized but then converges to a minimum, similar to the spiral dataset after observing enough timepoints. The first epoch is ignored, and all the calculations are made after the first 50 timepoints.

Once trained, instead of using the mean squared error as used in the training, we use the more general r-squared metric in order to test how well the trajectories originating at the predicted initial condition fit the future datapoints. Unlike the mean squared error metric, the r-squared metric would generalize even when the number of brain regions are changed or under different normalizations allowing it to be more compatible with future algorithms that test short term predictability. In Figure 3.6 right, we also tested how well the measure generalizes from testing once every epoch (50 timepoints) to testing every timepoint as well as the effects of computing the r-squared over a batch of data consisting of 60 individuals vs testing each individual at a time. The accuracy at the 3rd timepoint from the initial condition is plotted for these four conditions (i.e the permutations of individual vs group and one timepoint vs all timepoints). There is no difference in the mean or the variance in testing at every timepoint vs testing on all timepoints. This is not surprising as the algorithm was developed to predict the correct the initial conditions at every timepoint of the timeseries and the experiment shows that it generalizes and performs relatively similarly on all timepoints. On the other hand, there is a difference in the variance but not the mean when comparing the group r-squared values vs the individual r-squared values. This can be explained as individual differences in fMRI are averaged out in the group metric. This shows that our approach might be sensitive for individual differences, but we use the group metric for the subsequent results as they are more robust and allow us to test our hypothesis on the differences between different BNM. Moreover, since we utilize only a group averaged structural matrix, we are more interested how well the BNM fit to the group than to any particular individual.

Supplementary Figure 5: Individual Variability and Generalization Across Time

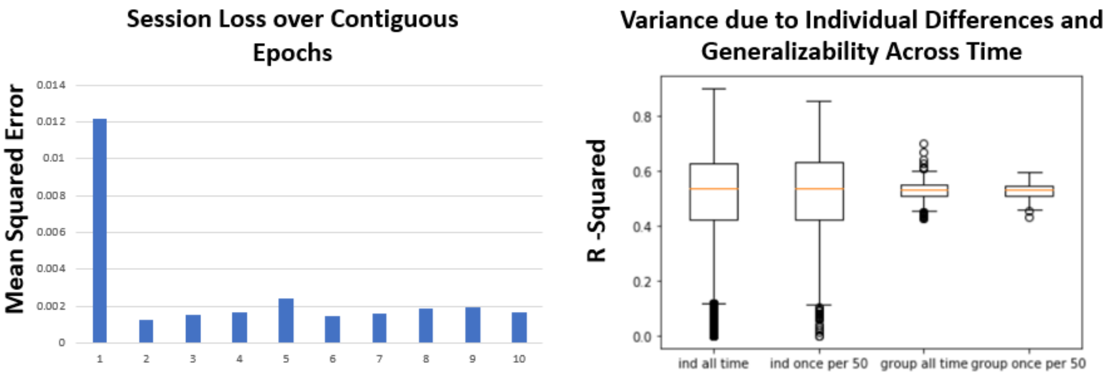

Left: The mean squared error calculated over each epoch of 50 timepoints from a continuous 1200 timepoints of fMRI data. The loss is constant after the first epoch which is much higher since the RNN hidden state is not properly initialized. The constant loss also suggests that after the first epoch the estimation of the initial condition is constant and has converged. The first epoch is not used in any of the subsequent estimates of evaluating the dynamical system. Right: The R-squared of a FRM (k = 0.9, σ = 0.3) is computed using 4 different methods. The first one (ind all time) evaluates the most number of tests, where the r-squared of each individual is calculated on every timepoint after the first epoch. The second one evaluates the r-squared of each individual fMRI once per epoch. The last two averages the r-squared across a batch of individuals at every timepoint and once per epoch. There is no difference in evaluating once per epoch or at every timepoint. There is a difference between the individual and group measures, which is expected as the group measure averages out the effect of individual variance. In our subsequent results we use the group measure, as our model does not take into individual differences in the structural matrix, and the group measure is more robust in evaluating the differences in the ODE which is what we are interested in.
